# Population games with instantaneous behavior and the Rosenzweig–MacArthur model

**DOI:** 10.1007/s00285-022-01821-4

**Published:** 2022-10-14

**Authors:** Emil F. Frølich, Uffe H. Thygesen

**Affiliations:** Department of Applied Mathematics and Computer Science - DTU Compute, Technical University of Denmark, Building 303B, Matematiktorvet, Kgs. Lyngby, Denmark

**Keywords:** Game theory, Population dynamics, Habitat choice, Population game, 49J40, 91A07, 91A22, 92D25, 92D50

## Abstract

How to determine the spatial distribution and population dynamics of animals are some of the key questions in ecology. These two have been coupled before, but there is no general method for determining spatial distributions based on instantanous behavior coupled with population dynamics. We propose modeling interacting populations with instantaneous habitat choice through mean-field games. By using the framework of variational inequalities, we are able to determine existence and uniqueness for habitat distributions of interacting populations, in both continuous and discrete habitats. With some additional restrictions, we are also able to show existence and uniqueness of fixed-points of the population dynamics along with spatial distributions. We illustrate our theoretical results by studying a Rosenzweig–MacArthur model where predators and consumers inhabit a continuous habitat. This study is conducted both theoretically and numerically. Analyzing the emergent dynamics is possible as viewing the system from the vantage point of variational inequalities allows for applying efficient numerical methods. The generality of our theoretical approach opens up for studying complex ecosystems, e.g. the impact of enrichment on spatial distributions in marine ecosystems.

## Introduction

Game theory is a natural tool to model the behavior of animals, who must respond to the behavior of other animals as well as complex and rapidly shifting environments. A classical application of game-theory is patch-choice models, where the ideal free distribution emerges to explain spatial distributions of populations (Cressman et al. 2004). A game theoretical approach has been fruitful in studying habitat choice in simple ecosystems under the assumption of static populations or simplifying the habitat to a few discrete patches (Cressman and Křivan 2010; Valdovinos et al. 2010). Real-life habitat choice consists of animals choosing where to forage in a continuous landscape, with varying intra-specific competition and external risk factors. Building models that can handle these challenges would represent a significant step forward in understanding natural systems (Morris 2003).

A population game is a system of interacting populations where each individual chooses the best strategy at every instant. Typically, this is the strategy that maximizes individual fitness. That is, population games generalize the ideal free distribution (Cressman et al. 2004). The single-species ideal free distribution is characterized by evolutionary stability, but stability in the multi-species case is more complex (Křivan et al. 2008). In the multi-species case evolutionary stability is not immediate when each animal optimizes their fitness. We refer to the ideal free distribution without stability assumptions as the simple ideal free distribution. When including behavior in population models using game-theory a common simplification is to assume that at least one payoff is linear in the choice of strategy (Krivan 1997). Linear models are suffficient to explain simple predator–prey dynamics with optimal behavior (Křivan 2007), but non-linear effects in natural systems are substantial (Gross et al. 2009).

A general model for population games based on fitness is set out in Vincent and Brown (2005) where optimal behavior is introduced by every population maximizing the per capita growth at every instant. This implicitly assumes monomorphic populations, where all individuals intrinsically act as one (Malone et al. 2020; Stump and Chesson 2017). The assumption of monomorphic populations is the typical approach to population games with instantaneous migrations (Křivan 2013; Vincent and Brown 2005), but it is well-known that this does not generalize the ideal free distribution and dramatically increases the per capita gain (Křivan et al. 2008). We propose a modification of the approach from Vincent and Brown (2005) in the vein of Cressman and Křivan (2010), based on individual optimization in the context of habitat selection. Rather than assuming a population where all individuals act in lockstep, we allow each anima to act independently with its risk-reward calculus affected by the population mean behavior (Fretwell 1969; Smith 1982; Cressman and Křivan 2010). Then the game at every instant game becomes a mean field game with multiple types, which leads to the simple ideal free distribution if the animals are optimizing their fitness.

We model instantaneous movement, but the underlying reality is that animals migrate between adjacent patches, e.g. through advection–diffusion dynamics (Cantrell et al. 2010). If population dynamics are sufficiently slow, then the migration dynamics which lead to the simple ideal free distribution are those which are evolutionarily stable (Averill et al. 2012; Cantrell et al. 2010), and even very basic migration dynamics lead to the simple ideal free distribution (Avgar et al. 2020). As such, populations at a population-dynamical equilibrium can be expected to follow a distribution where each individual has optimal fitness (Cantrell et al. 2007; Cressman and Křivan 2010), which in this case is zero. When the population dynamics and migratory time-scales are sufficiently decoupled, the migration dynamics which lead to the simple ideal free distribution are also evolutionarily stable (Cantrell et al. 2020; Cressman and Křivan 2006), even when predators do not directly optimize their own fitness (Avgar et al. 2020). Therefore a wide range of natural systems can be modeled by coupling population dynamics to optimal patch distribution. Currently there is no general approach to do so, but we introduce an approach based on mean-field games and optimization.

We essentialy unite the two parallel tracks which mean-field games have followed since their inception. One track is in mathematical biology through the ideal free distribution and habitat selection games (Fretwell 1969; Parker 1978; Cressman et al. 2004; Křivan et al. 2008; Cressman and Křivan 2010; Broom and Rychtár 2013), and the other in mathematical optimization based directly on anonymous actors (Lasry and Lions 2007; Aumann 1964; Blanchet and Carlier 2016). The main focus in the game-theoretically focused ecological work has been studying specific families of games in depth (Broom and Rychtár 2013), while the focus in mathematical optimization has been in establishing uniqueness and existence of Nash equilibria through the toolbox of variational inequalities (Karamardian 1969; Gabay 1980; Nabetani et al. 2011).

Using the theory of variational inequalities, we show that population games based on individual optimization have a unique equilibrium under very mild assumptions. Our approach allows us to handle both continuous and discrete strategy spaces, but more technical assumptions are required for existence in the continuous setting. The simple ideal free distribution emerges as a special case of our approach, providing a compelling argument for the mean-field approach. By working with variational inequalities, we can generalize the classical definition of a multi-species evolutionary stable state to the continuous setting (Cressman et al. 2001). We demonstrate our approach by applying it to a Rosenzweig–MacArthur system with intraspecific predator competition in continuous space modeling a marine ecosystem. We modify the system so both predators and consumers have instantaneous optimal behavior based on maximizing the individual growth rate. We show that the Rosenzweig–MacArthur system with optimal behavior satisfies the criteria for existence and uniqueness of equilibria as a population game, and perform numerical experiments to see the effect of the carrying capacity and competition on the system.

In addition to our theoretical advances, we implement a simple and efficient numerical method of finding Nash equilibria and equilbria of population games. The numerical method is applied to the behaviorally modified Rosenzweig–MacArthur system. We examine the population dynamics through a phase portrait, where they appear to be asymptotically stable. We study the population levels and spatial distribution at equilibrium as a function of the carrying capacity and intraspecific predator competition. With optimal behavior, increased competition causes a drastic change in behavioral patterns for consumers and predators and an increase in consumer populations with a very low impact on predator populations. Increasing carrying capacity causes both predator and consumer populations to increase, while consumers move towards more cautious behavior.

The paper is organized as follows: We start with the general setting. After building the general setting, we introduce the machinery of variational inequalities in the context of game theory. Here we show the general uniqueness and existence results. We proceed to study the concrete Rosenzweig–MacArthur model, showing existence and uniqueness of the Nash equilibrium and population equilibrium. We analyze the results, and discuss the implications of both numerical and theoretical results.

## Population games based on habitat choice

We build the general setting piece-by-piece, from the environment to the foraging strategies. First we define the environment, then we introduce the mean-field setting, as it is necessary to handle the strategy of an entire population. With this in place, we can give an exact definition of a population game in our sense. Once we have laid the building blocks for our setting, we show that mean-field games generalize the ideal free distribution.

We envision a setting with *M* different unstructured populations of animals co-existing in an environment, each with biomass $N_i$. We only model behavior as patch choice, excluding e.g. mating behavior. The distribution of population *i* in the environment is described by $\overline{\sigma }_i$. More rigorously, we assume that the environment is a probability space $(X,\mu )$. Modeling the environment as a probability space allows us to model habitats which are continuous, discrete and mixtures thereof in the same context. As an example, bats forage over a continuous area while the caves where they rest are discrete and disconnected (Collet 2019). We model that the populations $N_i$, $i\in \{1,\dots ,M\}$ are large compared to a single individual. This allows us to consider the population as continuous, consisting of infinitely many individuals. We assume that the population dynamics depend both on the distributions and the population sizes:

1 $$\begin{aligned} {\dot{N}}_i = N_i f_i((N_j\overline{\sigma }_j)_{j=1}^M) \end{aligned}$$

That is, we consider population dynamics which can be described by a Kolmogorov model.

We suppose that the migration dynamics happen on a faster time-scale than the population dynamics, as is seen with e.g. vertical migrations in marine ecosystems (Iwasa 1982). This slow-fast dynamic allows us to model the migrations as instantaneous, with each individual picking the optimal foraging ground at every instant (Křivan 2013; Cressman and Křivan 2006).

We assume that every animal has an area where it forages at every instant. For an animal of type *i* this is described by a probability distribution $\sigma _i$ over the environment *X*. We require that the distribution $\sigma _i$ is absolutely continuous with respect to the measure $\mu$. In an abuse of notation, we will denote this density by $\sigma$. We denote the space of probability densities over *X* with respect to $\mu$ by $P_{\mu }$. We suppress *X* for notational brevity. By requiring absolute continuity with respect to the base measure we remove degenerate Nash equilibria e.g. Dirac-type distributions in a continuous setting, avoiding for example all gazelles stacked exactly at a single point in space. We hereby generalize both the continuous and discrete approach to habitat selection (Fretwell 1969; Broom and Rychtár 2013; Thygesen and Patterson 2018).

### Foraging strategies and mean-field

In habitat choice games an animal faces the essential choice of where to forage, weighing risk and reward. Hence the density $\sigma _i$ describing where it forages is a strategic choice. As we assume instantaneous migrations and perfect information, an animal of type *i* faces the foraging choices of all other inhabitants. Modeling the influence of the foraging choices necessitates the introduction of the mean-field strategy, $\overline{\sigma }_j$ for type *j*. The mean-field strategy $\overline{\sigma }_j$ is the average strategy of all individuals of type *j*. As a consequence, we can describe the foraging presence from type *j* at a point $x\in X$ by $N_j \overline{\sigma }_j(x)$.

The choice of optimal foraging strategy $\sigma _i^*$ for an animal of type *i* is a trade-off based on the presence of competitors, predators and prey. When considering animal populations, finding the optimal behavior for all individuals simultaneously quickly becomes infeasible. For this reason, we need to simplify the problem. This is where mean-field games come into play. The fundamental idea behind a mean-field game is that in a sufficiently large population, the decision of a single individual has no discernible impact on the average behavior of the population. In this case we can decouple the behavior of an individual and the mean behavior of the population, and assume that an individual plays against the average behavior of the population. That is, an individual plays the field (Smith 1982). The fundamental assumption in the mean-field game that we consider is that the populations consist of infinitely many individuals acting instantaneously and independently so the choice of a single individual does not change the mean-field strategy (Aumann 1964)

The mean density of competitors, predators and prey at a point *x* is described by $N_j \overline{\sigma }_j(x)$. We capture this trade-off for for each individual in a payoff function $U_i(\sigma _i, (N_j \overline{\sigma }_j)_{j=1}^M)$. The payoff $U_i$ we have in mind is the instantanenous growth of an individual, i.e. individual fitness. This is given by the difference between the instantaneous per capita reproduction and mortality for an individual in Eq. 1. When using the individual fitness as payoff, the Nash equilibria we find should be the same as simple ideal free distributions.

Given that each type *j* is distributed according to $\overline{\sigma }_j$, the goal of a single animal of type *i* is finding the optimal strategy $\sigma _i^*$ by playing the field at each instant such that

2 $$\begin{aligned} \sigma ^*_i \in {{\,\mathrm{argmax}\,}}_{\sigma _i \in P_{\mu }} U_i(\sigma _i, (N_j\overline{\sigma }_j)_{j=1}^M) \end{aligned}$$

Whether such a Nash equilibrium exists is well established when no additional regularity is imposed on the probability distributions (Glicksberg 1952), we will tackle the general problem of existence later. At the Nash equilibrium of a mean-field game every individual of type *i* follows the same strategy $\sigma _i^*$, (Lasry and Lions 2007; Aumann 1964). Heuristically, this is due to interchangeability as if any individual of type *i* gains by deviating from $\sigma _i^*$, any one of them would also gain from making the same deviation, hence doing so. Therefore if all individuals follow the optimal strategy, they follow the same strategy. This allows us to go from the individual-level optimization to the Nash equilibrium in Eq. (2).

Using $^N$ to denote the Nash equilibrium, a mean-field equilibrium $\overline{\sigma }_i^N$ is a solution to the equation:

3 $$\begin{aligned} \overline{\sigma }_i^N = \left( {{\,\mathrm{argmax}\,}}_{\sigma _i \in P_{\mu }} U_i(\sigma _i, (N_j \overline{\sigma }_j)_{j=1, j\ne i}^M, N_i \overline{\sigma }_i^N) \right) \end{aligned}$$

A solution is guaranteed to exist by the results of Glicksberg (1952). Hence a Nash equilibrium of a game with *M* interacting populations is a solution to the system of equations:

4 $$\begin{aligned} \begin{aligned} \overline{\sigma }_1^N = \left( {{\,\mathrm{argmax}\,}}_{\sigma _1 \in P_{\mu }} U_1(\sigma _1, (N_j \overline{\sigma }^N_j)_{j=1}^M) \right) \\ \vdots \\ \overline{\sigma }_M^N = \left( {{\,\mathrm{argmax}\,}}_{\sigma _M \in P_{\mu }} U_M(\sigma _M, (N_j \overline{\sigma }^N_j)_{j=1}^M) \right) \end{aligned} \end{aligned}$$

This system of equations looks intractable, but in the next section we will see that in many cases it can actually be solved using the toolbox of variational inequalities. Introducing Eq. (4) allows us to define a population game exactly.

#### Definition 1

A population game consists of *M* unstructured populations with each population having a biomass of size $N_i$ with dynamics given by Eq. (1). Each individual of type *i* has a payoff function $U_i(\sigma _i, (N_j \overline{\sigma }_j)_{j=1}^M))$. Migrations are instantanenous, and at every instant the populations are distributed according to the mean-field Nash equilibrium Eq. (4), $\overline{\sigma }_i^N$.

The canonical example of Definition 1 is the case where the payoff functions $U_i$ are given by the individual fitness. The Nash equilibrium Eq. (4) becomes a situation where all individuals of each type have the same fitness and do not gain from deviating Eq. (4), i.e. the simple ideal free distribution (Fretwell 1969). We repeat the caveat that this version of the ideal free distribution does not incorporate any stability criteria (Křivan et al. 2008). For this reason we refrain from using the terminology "the ideal free distribution" and instead refer to Eq. (4) as the Nash equilibrium of a mean-field game or the simple ideal free distribution. We will give a definition of the multi-species ideal free distribution once we have introduced the entire framework of variational inequalities and their coupling with Nash equilibria.

Though we focus on population games with the individual fitness as payoff function, an appeal of the mean-field approach is that it allows general payoff functions. As an example, the impact of cooperation in a spatially extended game can be investigated by using a mean-field approach (Antonov et al. 2021).

## Nash equilibria and variational inequalities

Calculating Nash equilibria, Eq. (4) is generally a hard problem. A fruitful approach to calculating Nash equilibria is via the theory of complementarity problems and variational inequalities (Karamardian 1969; Nabetani et al. 2011). We unite the approach of variational inequalities and mean-field games which allows us to characterize a situation that guarantees uniqueness and existence of Nash equilibria in population games (Definition 1), and the existence of fixed-points of these games.

As in Sect. 2, our habitat is a probability space $(X,\mu )$. We have *M* different animal types coexisting with individual payoff-functions $U_i$. The simplest example our framework needs to handle is that of a single type with population *N* inhabiting *X* with following a distribution with density $\sigma$. The pointwise encounter rate of an individual following the strategy $\sigma$ with the entire population also following the strategy $\sigma$ is $N\sigma (x)^2$. The expected total encounter for an individual with its conspecifics is then

5 $$\begin{aligned} N\int _X \sigma ^2 d\mu \end{aligned}$$

and this quantity must be finite. This motivates that the appropriate setting for our work is the space $L^2(X)$.

### Definition 2

Define the real Hilbert space $H=L^2(X)$, where *X* is a probability space. Define $H_+ \subset H$ as the a.e. positive functions in *H*.

### From Karush–Kuhn–Tucker to complementarity

In order to find the Nash equilibrium at every instant in a population game, we need to solve Eq. (4). We recall the setup of the *M*-player mean field game, now restricted to *H*. Assume we have *M* different types of animals, with payoff functions $U_i$, and strategies $\sigma _i$, with corresponding mean-field strategies $\overline{\sigma }_i$. Before we proceed, we need to recall a simple version of the Karush–Kuhn–Tucker (KKT) conditions that we need. We denote the identity operator on *H* by $1_H$. For the full version of the KKT conditions, see e.g. Deimling (2010).

#### Theorem 1

A minimum $x^*$ of a Gateaux differentiable function *f* in $P_{2,\mu } \subset L^2(X)$ satisfies the necessary condition that there exists an element $\nu \in H^+$ and a scalar $\lambda \in {\mathbb {R}}$ such that:

6 $$\begin{aligned} \begin{aligned} f(x^*) + \nu&= 1_H \lambda \\ \left\langle x^*,\nu \right\rangle&= 0 \end{aligned} \end{aligned}$$

The condition $\left\langle x^*,\nu \right\rangle = 0$ is described as the complementary slackness conditions, and the requirements that $x^* \ge 0$ and $\int x^* d\mu = 1$ are the primal conditions. The variable $\lambda$ is a Lagrange multiplier, and $\nu$ is typically referred to as a slack variable.

The Nash equilibrium of the game specified by the family $(U_i)$ corresponds to finding a system $\sigma _i^*$ satisfying the KKT conditions simultaneously for every $U_i$, with $\overline{\sigma }=\sigma$ as in Eq. (3). The total criterion for a Nash equilibrium of a mean-field game Eq. (4) is:

7 $$\begin{aligned} \begin{aligned} \nabla _{\sigma _i}U_i((\sigma _j)_{j=1}^M) \mid _{\sigma _i = \overline{\sigma }_i} + \nu _i - \lambda _i \cdot 1_H = 0 \\ \left\langle \sigma _i,\nu _i \right\rangle = 0 \\ \nu _i \in H_+,~\sigma _i \in H_+ \\ \int _X \sigma _i d\mu (x)- 1 = 0 \end{aligned}\nonumber \\ \end{aligned}$$

Remark that the last two conditions are equivalent to $\sigma \in P_{\mu } \cap H$. This motivates the definition:

#### Definition 3

Assume we have a probability space $(X,\mu )$. Consider the space of square-integrable functions $H=L^2(X,\mu )$ and space $P_{\mu }$ of probability densities over *X*. Define the space $P_{2,\mu }=H \cap P_{\mu }$ consisting of square-integrable probability densities.

Solving the system in Eq. (7) is highly non-trivial, but it turns out that reinterpreting the problem is helpful. Finding Nash equilibria by interpreting the problem as a complementarity problem is one of the the original solutions to the hardness of finding Nash equilibria (Karamardian 1969). It turns out that the set of equations in Eq. (7) is very close to being a complementarity problem, but first we need to introduce the notion (Hadjisavvas et al. 2006, p. 507).

#### Definition 4

Let *H* be a real Hilbert space, and $K \subset H$ be a closed convex cone. Define $K^* = \{ x \in H: \left\langle x,y \right\rangle \ge 0, \quad \forall y\in K\}$. Assume $T:K \rightarrow H$. The complementarity problem *CP*(*T*, *K*) is the problem of finding an element *x* such that

8 $$\begin{aligned} \begin{aligned} \left\langle x,Tx \right\rangle = 0 \\ Tx \in K^*, \quad x\in K \end{aligned} \end{aligned}$$

In Definition 4 we recover the notion of a linear complementarity problem if *T* is affine.

With Definition 4 we can write Eq. (7) as an equivalent family of complementarity problems. Introduce $K = H_+ \oplus {\mathbb {R}}$, with $K^* = H_+ \oplus \{0\}$ and define

9 $$\begin{aligned} T(\sigma _i, \lambda _i) = (-\nabla _{\sigma _i} U_i + \lambda _i \cdot 1_H \mid _{\sigma _i = \overline{\sigma }_i}, 0) \end{aligned}$$

Then the equations in Eq. (7) can be recast as finding $(\sigma _i,\lambda _i) \in K$ such that:

10 $$\begin{aligned} \begin{aligned} \left\langle T(\sigma _i,\lambda _i),(\sigma _i,\lambda _i) \right\rangle = 0 \\ T(\sigma _i,\lambda _i) \in K^* \end{aligned} \end{aligned}$$

Which, when writing out the definition of *T*, becomes:

11 $$\begin{aligned} \begin{aligned} \left\langle -\nabla _{\sigma _i} U_i \mid _{\sigma _i = \overline{\sigma }_i} + \lambda _i \cdot 1_H,\sigma _i \right\rangle + \left\langle 0,\lambda _i \right\rangle = 0 \\ \left( -\left( \nabla _{\sigma _i} U_i \mid _{\sigma _i = \overline{\sigma }_i} + \lambda _i \cdot 1_H \right) , 0 \right) \in K^* \end{aligned} \end{aligned}$$

There are dedicated tools available allowing for fast numerical resolution of complementarity problems in finite dimensions (Acary et al. 2019; Dirkse and Ferris 1995), which can be applied after suitable discretization of the problem. There is still the problem of establishing existence and uniqueness of the solution to this complementarity problem, which is generally hard, (Hadjisavvas et al. 2006).

### Results on variational inequalities

Before we can proceed with the main theme of the article, we recount some results on existence and uniqueness of variatinoal inequalities, which also show their general utility in optimization. We define a variational inequality:

#### Definition 5

Let *H* be a real Hilbert space and $K\subset H$ be a non-empty convex subset of *H*. Let $T: K \rightarrow H$. The variational inequality *VI*(*T*, *K*) is the following system for $x\ne y$:

12 $$\begin{aligned} x \in K, \left\langle y-x,Tx \right\rangle \ge 0, \quad \forall y \in K \end{aligned}$$

The relationship between variational inequalities and complementarity problems is captured in (Hadjisavvas et al. 2006, Proposition 12.1):

#### Proposition 1

Let $K\subset H$ be a convex cone, and $T: K \rightarrow H$. Then the variational inequality *VI*(*T*, *K*) is equivalent to the complementarity problem *CP*(*T*, *K*).

The solutions to a variational inequality are not generally unique, but with certain restrictions on *T* the solutions become unique.

#### Definition 6

The function $T: K \rightarrow H$ is strictly pseudomonotone if for every pair $x\ne y$ we have

13 $$\begin{aligned} \left\langle x-y,T(y) \right\rangle \ge 0 \Rightarrow \left\langle x-y,T(x) \right\rangle > 0 \end{aligned}$$

Likewise, the function *T* is pseudomonotone if for every pair $x\ne y$ we have:

14 $$\begin{aligned} \left\langle x-y,T(y) \right\rangle \ge 0 \Rightarrow \left\langle x-y,T(x) \right\rangle \ge 0 \end{aligned}$$

Which enables the uniqueness result:

#### Theorem 2

(Lemma 12.3, p. 516, (Hadjisavvas et al. 2006)) Let $K\subset H$ be a non-empty subset of *H*. If *T* is a strictly pseudomonotone function, then the problem *VI*(*T*, *K*) has at most one solution.

Strict pseudomonotonicity is related to strict monotonicity, in that every strictly monotone function is also strictly pseudomonotone. A natural question is how strictly pseudomonotone arise, and they arise from a corresponding generalization of strict convexity.

#### Definition 7

Let $\varOmega \subset H$ be an open subset of *H*, and let $f:\varOmega \rightarrow {\mathbb {R}}$ be Gateaux-differentiable. The function *f* is strictly pseudoconvex if

15 $$\begin{aligned} \left\langle y-x,(\nabla f)(x) \right\rangle \ge 0 \Rightarrow f(y) > f(x) \end{aligned}$$

Where a strictly convex function has a strictly monotone derivative, a variant holds for strictly pseudoconvex functions which have strictly pseudomonotone derivatives. Hence minimizing a differentiable strictly pseudoconvex *f* function over a convex set *K* is equivalent to solving the variational inequality (Hadjisavvas et al. 2006, P. 521)

16 $$\begin{aligned} x \in K, ~ \left\langle (\nabla f)(x),x-y \right\rangle \ge 0, \forall y\in K \end{aligned}$$

Having given a criterion for uniqueness, the next question is whether a solution exists at all. The existence of solutions to a variational inequality given by a pseudomonotone function can be determined by a simple criterion (Maugeri and Raciti 2009, Theorem 3.4), which we abridge:

#### Theorem 3

Let *K* be a closed convex set and $A: K \rightarrow H$ a pseudo- monotone map which is continuous on finite dimensional subspaces of *H*. A variational inequality $\left\langle A(x),y-x \right\rangle$ has a solution if and only if There exists a point $u_0 \in K$ such that the set

17 $$\begin{aligned} \{v \in K: \left\langle A(v),v-u_0 \right\rangle < 0\} \end{aligned}$$

is bounded. This provides us with a testable criterion for whether a variational inequality admits a solution.

#### Remark 1

Boundedness of *K*, or more precisely weak compactness, also ensures that *VI*(*T*, *K*) has a solution in *K* (Hadjisavvas et al. 2006, Theorem 12.1, P. 510). This also ensures existence of solutions to variational inequalities in the finite-dimensional case.

Intuitively, the criterion in Theorem 3 states that as long as there a direction where *A*(*v*) becomes positive eventually, there exists a solution to the variational inequality in *K*. Or, on a more formal level, what the criterion says is that instead of *K* being weakly compact, it is sufficient that $\left\langle A(v),v-u_0 \right\rangle$ changes sign on weakly compact set. In practice this criterion should always be satisfied in a population game, as a negative density dependence should eventually outweigh any gain from clumping as an infinite concentration should not be advantageous.

Though strictly pseudomonotone functions initially arise as gradients of strictly pseudoconvex functions, they can be much more general. Checking whether a function is strictly pseudomonotone from the definitions can also be hard in practice, hence we state another characterization of strict pseudomonotonicity for differentiable functions.

#### Lemma 1

Let *K* be a convex subset of *H*. A function $f: K \rightarrow {\mathbb {R}}$ is strictly pseudomonotone if the following implication holds for any $x,h \in K$:

18 $$\begin{aligned} \left\langle f(x),h \right\rangle = 0 \Rightarrow \left\langle (\nabla _x f(x))h,h \right\rangle > 0 \end{aligned}$$

A proof can be found in Hadjisavvas et al. (2006, Proposition 2.8, p.96)

### The Nash equilibrium as a variational inequality

We have recast the problem of finding a Nash equilibrium to a complementarity problem, which allows for numerical resolution. To establish existence and uniqueness, we need to use the relationship between complementarity problems and variational inequalities. We will show that in case the payoff-functions $$U_i $$ are sufficiently nice, the machinery of variational inequalities can be applied to show existence and uniqueness of the Nash equilibrium.

We can now turn the problem finding a Nash equilibrium into a variational inequality. Consider the problem as stated in Eq. (10). This is a complementarity problem over the convex cone $H_+ \oplus {\mathbb {R}}$. Hence it is equivalent to a variational inequality over the same convex cone with *T* as in Eq. (10) by Proposition 1. The equivalent variational inequality becomes that of finding a pair $\sigma _i, \lambda _i$ such that:

19 $$\begin{aligned} \left\langle T(\sigma _i,\lambda _i),(\sigma _i' -\sigma _i,\lambda _i'-\lambda _i) \right\rangle \ge 0, \quad \forall (\sigma _i', \lambda _i') \in K,~(\sigma _i', \lambda _i') \ne (\sigma _i, \lambda _i) \end{aligned}$$

Recalling the definition of *T*, $T=(-\nabla _{\sigma _i} U_i \mid _{\sigma _i = \overline{\sigma }_i} - \lambda _i, 0)$, we see the second coordinate is identically zero. Hence solving Eq. (19) is equivalent to solving

20 $$\begin{aligned} \begin{aligned} \left\langle -\nabla _{\sigma _i} U_i \mid _{\sigma _i = \overline{\sigma }_i} - \lambda _i,\sigma _i' -\sigma _i \right\rangle&\ge 0 \quad \forall \sigma _i' \in K,~\sigma _i'\ne \sigma _i \\ \left\langle -\nabla _{\sigma _i} U_i \mid _{\sigma _i = \overline{\sigma }_i},\sigma _i' -\sigma _i \right\rangle - \left\langle \lambda _i,\sigma _i' -\sigma _i \right\rangle&\ge 0 \quad \forall \sigma _i' \in K,~\sigma _i'\ne \sigma _i \end{aligned} \end{aligned}$$

If we constrain the solution set to the convex set $P_{2,\mu }$ where it must lie due to the Lagrange multiplier, both $\sigma _i$ and $\sigma _i'$ integrate to 1, therefore the term $\left\langle \lambda _i,\sigma _i' -\sigma _i \right\rangle$ vanishes. Hence solving Eq. (20) over *K* is equivalent to solving the variational inequality:

21 $$\begin{aligned} \left\langle -\nabla _{\sigma _i} U_i \mid _{\sigma _i = \overline{\sigma }_i},\sigma _i' -\sigma _i \right\rangle \ge 0, \quad \forall \sigma _i' \in P_{2,\mu },~\sigma _i'\ne \sigma _i \end{aligned}$$

We can now state the problem of finding the Nash equilibrium Eq. (4) as finding the solution of a variational inequality.

#### Definition 8

(Nash equilibrium as variational inequality) Defining

22 $$\begin{aligned} dU = \begin{pmatrix} \nabla _{\sigma _1} U_1 \mid _{\sigma _1 = \overline{\sigma }_1}\\ \vdots \\ \nabla _{\sigma _N} U_N \mid _{\sigma _N = \overline{\sigma }_N} \end{pmatrix} \end{aligned}$$

the problem of determining the Nash equilibrium Eq. (4) is the variational inequality of finding a vector $S = (\sigma _i)_{i=1}^M$ such that:

23 $$\begin{aligned} \left\langle -dU(S),W-S \right\rangle \ge 0 \quad \forall W\in (P_{2,\mu })^M~,W\ne S \end{aligned}$$

with $P_{2,\mu }$ as defined in Definition 3

With Definition 6 in hand, we can finally give sufficient criteria for existence and uniqueness of the Nash equilibrium of the game specified in Eq. (4).

#### Theorem 4

Consider a game with *M* players with payoff functions $U_i$ such that the total operator $-dU$ from Definition 8 is strictly pseudomonotone. Assume the strategies $\sigma _i$ are in $P_{2,\mu }$. The game has a unique Nash equilibrium if $-dU$ as in Definition 8 satisfies the criterion in part (2) of Theorem 3 or *H* is finite dimensional.

#### Proof

By Theorem 2 any Nash equilibrium will be unqiue since *dU* is strictly pseudomonotone. So if the solution exists, it is unique. By assumption Theorem 3 gives existence of a solution of $VI(dU,P_{2,\mu }^M)$ in case *H* is infinite dimensional. If *H* is finite-dimensional then $P_{2,\mu }^M$ is compact and there exists a solution by Remark 1. $\square$

With Theorem 4, we can show that there exist unique fixed points of population games where *dU* is strictly pseudomonotone and the vector fields specifying the population dynamics are sufficiently regular.

#### Theorem 5

We have a population game as Definition 1 with *M* populations of size $N_i$, payoff functions $U_i(\sigma _i, (N_j \overline{\sigma }_j)_{j=1}^M)$ and dynamics given by $f_i((N_j \overline{\sigma }_j)_{j=1}^M))$:

24 $$\begin{aligned} \dot{N_i} = N_i f_i \end{aligned}$$

Assume that the that the set of stationary points of the population dynamics is uniformly bounded in $(\sigma _i)_{i=1}^M$, and that the stationary points can be described by a continuous function $\varPhi :P_{2,\mu }^M \rightarrow {\mathbb {R}}_+^M$. Let $-dU = (\nabla _{\sigma _i} U_i \mid _{\sigma _i = \overline{\sigma }_i})$ be strictly pseudomonotone and satisfy the criterion of Theorem 3. Then the population game has a fixed point. If further the system $f_i$ defines a pseudomonotone operator $F:{\mathbb {R}}_+^M \rightarrow {\mathbb {R}}_+^M$ with $F=(f_1, \dots , f_M)$, the fixed-point is unique.

#### Proof

The game specified by the family $(U_i)_{i=1}^M$ defines a variational inequality problem over $P_{2,\mu }^M$ with operator $-dU$. This variational inquality has a unique solution for each $x\in {\mathbb {R}}_+^M$, due to the existence and uniqueness of the solution by Theorem 4. This solution defines a continuous a function from ${\mathbb {R}}_+^M$, denoted *G*, where $G: {\mathbb {R}}_+^M \rightarrow P_{2,\mu }^M$ (Barbagallo and Cojocaru 2009, Theorem 4.2).

Finding a fixed point of the dynamical system along with a Nash equilibrium then corresponds to finding a fixed point of the mapping $\varPhi \circ G: {\mathbb {R}}_+^M \rightarrow {\mathbb {R}}_+^M$. Since the set of stationary points is assumed bounded, *G* has compact range, and $\varPhi \circ G$ has compact image. Therefore $\varPhi \circ G:{\mathbb {R}}_+^M \rightarrow {\mathbb {R}}_+^M$ has a fixed point $(x_1^*, \dots , x_m^*)$ by Schauder’s fixed point theorem (Granas and Dugundji 2003, Theorem 3.2, p. 119).

We can conclude that a fixed-point exists, hence a combined Nash and population equilibrium.

To show uniqueness, we need to shift perspectives. We are searching for zeros of the system $f_i$, i.e. solutions of the variational inequality $VI(F, {\mathbb {R}}_+^M)$ constrained by the fact that the system of $\sigma _i$ constitute a Nash equilibrium, i.e. they need to solve the variational inequality $VI(-dU,P_{2,\mu }^M)$. This is an example of a so-called bi-level variational inequality. As we have already established existence, the strict pseudomonotonicity of $-dU$ and pseudomonotonicity of *F* give us uniqueness of the solution (Chen et al. 2014). This shows the desired result. $\square$

### The ideal free distribution

Having introduced the framework of variational inequalities allows us to connect with the ideal free distribution. As noted in the introduction, the ideal free distribution is classically defined as emerging from playing the field in single-species habitat selection games (Fretwell 1969). As such, the ideal free distribution is informally characterized by no individual gaining anything from moving from their spot in a habitat selection game. This definition, while perfectly suitable for single-species games is insufficient for the multi-species case. A stability requirement should also be introduced so a small deviation from the ideal free distribution will not change the overall distribution and such that best-response dynamics converge to the ideal free distribution (Křivan et al. 2008). The ideal free distribution can also be expanded to incorporate population dynamics (Cressman and Křivan 2010), but we refrain from going in this direction here as it would bring us too far afield. As in Sect. 2 we consider *M* populations with mean-field strategies $(\overline{\sigma }_i)_{i=1}^M$, individual strategies $(\sigma _i)_{i=1}^M$ and individual payoff functions $U_i$. We assume that we have a population game with a total operator $-dU$ Definition 8, with components $-dU_i$.

We generalize the definition of Křivan et al. (2008) and go with a rather restrictive definition of the multi-species ideal free distribution which ensures stability. It is typically posed as a result that the ideal free distribution is an evolutionarily stable strategy (ESS), but we take it as a part of the definition. We introduce the notion of evolutionary stable strategies based on the definition on evolutionary stable states using variational inequalities (Migot and Cojocaru 2021). For simplicity, we do not take weakly evolutionary stable strategies into account but concern ourselves with the strict case.

In Cressman et al. (2004) the notion of an *M*-species evolutionarily stable strategy is introduced, which is equivalent to the ideal free distribution defined in terms of best responses, Křivan et al. (2008, Section 3.3).

#### Definition 9

A set of strategies $(\overline{\sigma }^N)_i)_{i=1}^M$ in an *M*-species population game is an evolutionarily stable strategy if invaders following the slightly perturbed strategies $(\sigma _i')^M_{i=1}$ do not have an advantage against the resident population. In our notation, implies that for at least one *i*, we have $U_i(\sigma _i',(\overline{\sigma }_j)_{j=1}^M)<U_i(\overline{\sigma }_i,(\overline{\sigma }_j)_{j=1}^M)$.

We can now relate strict pseudomonotonicity and evolutionary stable strategies, which motivates that strict pseudomonotonicity is the correct notion to look for in population games, apart from the uniqueness properties.

#### Theorem 6

Given a population game with payoff functions $(U_i)_{i=1}^M$ with total operator $-dU$, if each component $-dU_i$ is strictly pseudomonotone, the Nash equilibrium $(\overline{\sigma }_i^N)_{i=1}^M$ is an evolutionarily stable strategy.

#### Proof

We wish to show that $U_i(\sigma _i',(\overline{\sigma }_j)_{j=1}^M)<U_i(\overline{\sigma }_i,(\overline{\sigma }_j)_{j=1}^M)$. As the in assumption in Definition 9 is that $\sigma _i'$ is a slight perturbation of $\overline{\sigma }_i$, we can equivalently show

25 $$\begin{aligned} \left\langle \overline{\sigma _i}^N-\sigma _i',-dU_i((\overline{\sigma }_j)_{j=1}^M) \right\rangle > 0 \end{aligned}$$

As we assume each $-dU_i$ is strictly pseudomonotone and that $\overline{\sigma }^N_i$ is the Nash equilibrium for the game defined by $U_i(\sigma ,\overline{\sigma })$, by definition of pseudomonotonicity any strategy $\omega$ different from $\overline{\sigma }^N_i$ satisfies the inequality:

26 $$\begin{aligned} \left\langle -dU_i(\overline{\sigma }_i),\omega -\overline{\sigma }_i \right\rangle > 0 \end{aligned}$$

which is exactly the criterion for evolutionary stability of the strategy $\overline{\sigma }_i$. $\square$

This allows us to define an *M*-species ideal free distribution.

#### Definition 10

A Nash equilibrium $(\overline{\sigma }^N_i)_{i=1}^M$ of an *M*-species population game with payoff-functions $U_i$ given by the individual fitness is an *M*-species ideal free distribution if the Nash equilibrium $(\overline{\sigma }^N_i)_{i=1}^M$ is an evolutionarily stable strategy.

This allows us to state the result which motivates that strict pseudomonotonicity is the correct notion to look for in population games, apart from the uniqueness properties.

#### Corollary 1

If $-dU$ and each component $-dU_i$ are strictly pseudomonotone, the Nash equilibrium $(\overline{\sigma }_i^N)_{i=1}^M$ in a population game is unique and an ideal free distribution.

#### Proof

The uniqueness of the Nash equilibrium follows from the strict pseudomonotonicity of $-dU_i$. As we assume each $-dU_i$ is strictly pseudomonotone the Nash equilibrium also constitutes an ESS by Theorem 6. $\square$

The strict pseudomonotonicity in Theorem 1 is also sufficient for asymptotic convergence of the replicator dynamics to the Nash equilibrium (Migot and Cojocaru 2021), providing additional motivation for the choice of strict pseudomonotonicity as the defining characteristic in population games. Our definition of an evolutionarily stable strategy is closely related to that of an evolutionarily stable state (Migot and Cojocaru 2021). If all components $-dU_i$ are strictly pseudomonotone as in Theorem 1 and not just a single one or a few, the resulting ESS is even stable in the sense that it can invade other states (Apaloo et al. 2009).

Having established the general results for population games based on habitat choice with instantaneous migrations and introduced the connection to the ideal free distribution, we are ready to apply the theory to a Rosenzweig–MacArthur system with fast adaptive behavior.

## Revisiting the Rosenzweig–MacArthur model

We consider a predator–prey system modeled as a Rosenzweig–MacArthur system where each individual consumer and predator seeks to maximize its growth at every instant, in the vein of Krivan and Cressman (2009). We represent consumer, respectively predator, per capita growth by $G_c$ and $G_p$. Likewise, we represent the per capita mortality by $M_c$ and $M_p$. We denote the growth and mortality rates of an individual by the superscript $^{ind}$. Defining the per capita dynamics $f_c = G_c - M_c$ and $f_p = G_p - M_p$, we can write the dynamical system for the population dynamics as:

27 $$\begin{aligned} \begin{aligned} \dot{N_c}&= N_c f_c \\ \dot{N_p}&= N_p f_p \end{aligned} \end{aligned}$$

The payoff functions for an individual consumer and predator are given by the individual growth rates $U_c,U_p$, and they are:

28 $$\begin{aligned} \begin{aligned} U_c(\sigma _c, N_c \overline{\sigma }_c, N_p\overline{\sigma }_p)&= G_c^{ind} - M_c^{ind} \\ U_p(\sigma _p, N_c \overline{\sigma }_c, N_p\overline{\sigma }_p)&= G_p^{ind} - M_p^{ind} \end{aligned} \end{aligned}$$

We consider a system with zooplankton as consumers $(N_c)$ and forage fish as predators $(N_p)$ in the water column, modeled as the interval [0, 100], with 0 as the top of the water column and 100 as the bottom. The choice of strategy is the depth at which to forage. Both forage fish and zooplankton have large populations, so it is reasonable to model this system as a mean-field game. We denote the mean strategies of the predator and consumer populations by $\overline{\sigma }_c$, and $\overline{\sigma }_p$. The productive zone of the water column, i.e. where zoo-plankton can find food, is near the top where sunlight allows phytoplankton to grow. Forage fish are visual predators, so their predation success is greatest near the top of the water column (Schadegg and Herberholz 2017). We model an arctic summer where there is constant sunlight which allows us to to ignore the influence of the day-night cycle. Both zooplankton and foraging fish populations in the arctic are mainly driven by the summer (Astthorsson and Gislason 2003; Mueter et al. 2016).

As zooplankton are olfactory foragers, we model that their growth rate $\beta _c$ is constant throughout the water column but the carrying capacity varies. We assume the zooplankton are not limited either by maximal consumption or handling (Kiørboe 2011), which coupled with the varying capacity leads to a logistic model for their growth. Summarizing, we assume that the maximal potential growth for a consumer from a location depends both on the absolute carrying capacity and how many consumers are already occupying the spot. We model the carrying capacity as $K_0 + K \phi$ where $K_0$ is the minimal carrying capacity, *K* is the varying capacity and $\phi$ is a probability density function. The per capita growth rate of a consumer becomes:

29 $$\begin{aligned} G_c(N_c, \overline{\sigma }_c) = \beta _c\left\langle \overline{\sigma }_c,1-N_c \frac{\overline{\sigma }_c}{K\phi + K_0} \right\rangle \end{aligned}$$

The mortality of the consumers is directly related to the growth of the predators, so we define the growth of the predators and then come back to the mortality of the consumers. Predator–prey interactions are fundamentally governed by the clearance or catch rate $\beta _p$ which describes the change in encounter rate from an increase in consumer or predator concentration. The encounter rate incorporates the light-dependent nature of forage fish, while incorporating that that there is still a minimal chance of catching prey without light. Concretly, we define:

$$\begin{aligned} \beta _p = \beta _{l} + \beta _0 \end{aligned}$$

where $\beta _l$ varies locally and $\beta _0$ is the minimal clearance rate. We define the maximal consumption rate for a predator $F_p$ as the reciprocal of the handling time of a predator $H_p$:

30 $$\begin{aligned} F_p = \frac{1}{H_p} \end{aligned}$$

The choice of maximal consumption rate as a parameter rather than handling time reflects that marine animals are rarely limited by handling (Schadegg and Herberholz 2017). We assume the maximal predator consumption rate is $H_p$, and the predators have a conversion efficiency of $\varepsilon$. Consumption events are assumed local, so the expected encounter rate between predators and prey is $N_c N_p \left\langle \beta _p \overline{\sigma }_p,\overline{\sigma }_c \right\rangle$. We assume that predators have a Type II functional response, and their consumption is limited by prey-capture and digestion rather than handling, which causes a non-linearity in the functional response as a function of the strategy (Kiørboe et al. 2018). This gives a per capita predator growth rate $G_p$:

31 $$\begin{aligned} G_p(N_p, \overline{\sigma }_p, N_c, \overline{\sigma }_c )= \varepsilon \frac{F_p\left\langle \beta _p N_c\overline{\sigma }_c,\overline{\sigma }_p \right\rangle }{F_p + \left\langle \beta _p \overline{\sigma }_c,\overline{\sigma }_p \right\rangle N_c} \end{aligned}$$

Having defined the growth rate of the predators allows us to define the per capita consumer mortality $M_c = \frac{N_p}{\varepsilon N_c}G_p$. Predator losses stems both from a metabolic loss $\mu _p$ and mortality from intraspecific predator competition, which we assume leads to a quadratic loss for predators as there is no satiation. We assume that predators losses from competition are greatest in the area where they are best specialized for hunting, since this is where they are best able to confront their con-specifics. Introducing a competition parameter *c*, the per capita predator mortality $M_p$ is:

32 $$\begin{aligned} M_p(N_p, \overline{\sigma }_p) = c \left\langle \overline{\sigma }_p,N_p\beta _p \overline{\sigma }_p \right\rangle + \mu _p \end{aligned}$$

Hence the population dynamics in Eq. (27) are a modified Rosenzweig–MacArthur system, where behavior of both consumer and predator populations has been incorporated. Having considered the population dynamics, we now proceed to the individual level.

### The instantaneous game

Following the exposition in Sect. 2 we model predator and consumer movement as instantaneous. We assume that each predator and consumer seeks to maximize their instantanous growth at every instant. As we have switched to focusing on the individuals, we have to distinguish between the strategy of an individual and the mean-field strategy of the population. Denote the strategies of a focal consumer and predator by $\sigma _c$ and $\sigma _p$ respectively. The growth of the focal individual depends on the mean-field strategies of both predators and consumers, and can be arrived at by analysing the expressions for $G_c,M_c$ and $G_p, M_p$ carefully, noting which terms depend upon individual choice and which are dependent on the mean-field strategy.

The growth $G_c^{ind}$ of an individual consumer depends on the choices of the consumer, while the available food depends on the spatial distribution of the entire population. Hence the growth of an individual consumer is:

33 $$\begin{aligned} G_c^{ind} = \beta _c\left\langle \sigma _c,1-N_c \frac{\overline{\sigma }_c}{K\phi + K_0} \right\rangle \end{aligned}$$

The loss from predation $(M_c)$ for an individual consumer is more complex. The risk of encountering a predator depends on the strategy of the focal consumer and the overall predator distribution, while the satiation of the predator depends on how many total consumers it encounters, hence the mean of the population behavior. Therefore the individual mortality of a consumer $M_c^{ind}$ becomes

34 $$\begin{aligned} M_c^{ind} = \frac{F_p \left\langle \beta _p \sigma _c,\overline{\sigma }_p \right\rangle N_p}{F_p + N_c\left\langle \beta _p \overline{\sigma }_c,\overline{\sigma }_p \right\rangle } \end{aligned}$$

Going to a focal predator, the growth $G_p^{ind}$ of an individual predator has the same expression as the per capita growth, since the satiation of an individual predator has does not depend on the behavior of the other predators.

35 $$\begin{aligned} G_p^{ind} = \varepsilon \frac{F_p \left\langle \beta _p \overline{\sigma }_c,\sigma _p \right\rangle N_c}{F_p + \left\langle \beta _p \overline{\sigma }_c,\sigma _p \right\rangle N_c} \end{aligned}$$

The individual predator mortality $M_p^{ind}$ depends on both the strategy of the individual predator and the distribution of the entire predator population.

36 $$\begin{aligned} M_p^{ind} = c \left\langle \sigma _p,N_p\beta _p \overline{\sigma }_p \right\rangle + \mu _p \end{aligned}$$

### Existence and uniqueness of Nash and population equilibria

In order to establish existence and uniqueness of the Nash equilibrium we show that the variational inequality defined by $-dU$ is strictly pseudomonotone and admits a solution. We start by showing that there is a unique Nash equilibrium for the cases where the predator and consumer respectively have constant behavior, i.e. $\sigma _i = 1,~i\in \{c,p\}$. First we need a small lemma to simplify the calculations.

#### Lemma 2

A function $g: P_{2,\mu } \rightarrow H$ is pseudomonotone if and only if $g+\lambda$ is pseudomonotone for any $\lambda \in {\mathbb {R}}$.

#### Proof

Consider $\left\langle g(x)+\lambda ,x-y \right\rangle =\left\langle g(x),x-y \right\rangle +\lambda \int x d\mu - \lambda \int y d\mu$ Using that $\int y d\mu = \int x d\mu = 1$, we arrive at $\left\langle g(x),x-y \right\rangle$. Hence the pseudomonotonicity of *g* and $g+\lambda$ are equivalent. $\square$

#### Proposition 2

For every pair of non-zero abundances $N_c,N_p$ we have: There is a unique mean-field Nash equilibrium in the Rosenzweig–MacArthur system where the consumers have adaptive behavior and predators have constant behavior $\sigma _p = 1$. Likewise, there is a unique Nash equilibrium in the Rosenzweig–MacArthur system where the predators have optimal behavior and the consumers have constant behavior $\sigma _c = 1$.

#### Proof

To show the uniqueness of the Nash equilibrium when the consumers have optimal behavior, consider $dU_c = \nabla _{\sigma _c} U_c \mid _{\sigma _c = \overline{\sigma _c}}$. Without loss of generality, we may assume $\overline{\sigma }_p=1$ as the difference may be absorbed in $\beta _p$. By Lemma 2 it suffices to show that $f = -dU_c + 1$ is strictly pseudomonotone. To de-clutter the calculations we set $\beta _c = 1$ in the following calculations, but the necessary changes for an arbitrary value are straightforward. For Lemma 1 assume $\left\langle f((\overline{\sigma _c})),h \right\rangle = 0$, then

37 $$\begin{aligned} \begin{aligned}&\left\langle f(x),h \right\rangle = 0 \\&\left\langle \sigma _c \frac{N_c}{K\phi + K_0} ,h \right\rangle + \frac{N_p F_p \left\langle \beta _p,h \right\rangle }{F_p + N_c\left\langle \beta _p,\overline{\sigma _c} \right\rangle } - \left\langle 1,h \right\rangle + \left\langle 1,h \right\rangle = 0 \end{aligned} \end{aligned}$$

Hence

38 $$\begin{aligned} \left\langle \sigma _c \frac{N_c}{K\phi + K_0} ,h \right\rangle = - \frac{N_p F_p \left\langle \beta _p,h \right\rangle }{F_p + N_c\left\langle \beta _p,\overline{\sigma _c} \right\rangle } \end{aligned}$$

Introducing $\langle x |$ as the functional defined from *x*, consider

$$\begin{aligned} H((\overline{\sigma _c},h)= \left\langle (\nabla f)((\overline{\sigma _c},\sigma _p))h,h \right\rangle \end{aligned}$$,

where $\left\langle f(\overline{\sigma _c}),h \right\rangle = 0$. We calculate $\nabla f$:

39 $$\begin{aligned} \nabla f = \begin{pmatrix} \frac{N_c}{K \phi + K_0} - \frac{F_p N_c \langle \beta _p | \langle \beta _p |}{(F_p + N_c\left\langle \beta _p,\overline{\sigma _c} \right\rangle )^2} \end{pmatrix} \end{aligned}$$

So

40 $$\begin{aligned} H(\overline{\sigma _c},h) = \left\langle \frac{N_c}{K \phi + K_0}h,h \right\rangle - \left\langle \frac{F_p N_c N_p\left\langle \beta _p,h \right\rangle \beta _p}{(F_p + N_c\left\langle \beta _p,\overline{\sigma _c} \right\rangle )^2}h,h \right\rangle \end{aligned}$$

Inserting Eq. (38) in Eq. (40), we arrive at

41 $$\begin{aligned} H(\overline{\sigma _c},h) = \left\langle \frac{N_c}{K \phi + K_0}h,h \right\rangle + \frac{N_c}{N_p F_p}\left( \left\langle \overline{\sigma _c} \frac{N_c}{K\phi + K_0},h \right\rangle \right) ^2 \end{aligned}$$

As $\frac{N_c}{K \phi + K_0}$ is strictly positive, we conclude that $H(\overline{\sigma _c},h)>0$. Therefore *f* is strictly pseudomonotone by Lemma 1. The situation for the predators is even simpler, since $-dU_p$ is strictly monotone, hence strictly pseudomonotone, so the Nash equilibrium is unique. The existence of the Nash equilibria follows from the proof of existence in Proposition 3. $\square$

#### Remark 2

In Proposition 2 we considered the single-species game where the constant behavior was a uniform distribution. The proofs for constant behavior different from the uniform distribution are the same, but are heavier in notation.

Having shown that each of the underlying mean-field games has a unique Nash equilibrium, we can consider the total game.

#### Proposition 3

The game defined by $U_c$ and $U_p$ has a unique Nash equilibrium for every non-zero pair $N_c,N_p$. Further, this Nash equilibrium constitutes an ideal free distribution.

#### Proof

By Remark 2 and Proposition 2, any Nash equilibrium of this game is an ideal free distribution as both single-species game are strictly pseudomonotone by Theorem 1. Again, to simplify the notational load in the calculations we set $\beta _c = 1$, but the changes to accomodate an arbitrary value are straight-forward. To show existence of a Nash equilibrium, we need to show that the variational inequality defined by the function

42 $$\begin{aligned} dU = \begin{pmatrix}-\nabla _{\sigma _c} U_c \mid _{\sigma _c = \overline{\sigma _c}} \\ -\nabla _{\sigma _p} U_p \mid _{\sigma _p = \overline{\sigma }_p}\end{pmatrix} \end{aligned}$$

satisfies the criteria of Theorem 3 and is strictly pseudomonotone. To reduce notational clutter we write $\sigma _c$ in place of $\overline{\sigma _c}$ and $\sigma _p$ in place of $\overline{\sigma _p}$ through the remainder of the proof. To show that there exists a solution, start by noting that for all $S\in H^2$, $S\mapsto -dU(S)$ is Lipschitz continuous, hence continuous on finite-dimensional subspaces, fulfilling the first criterion of Theorem 3. For the second criterion, consider

43 $$\begin{aligned} \left\langle -dU(\sigma _c,\sigma _p),(\sigma _c-1, \sigma _p-1) \right\rangle \end{aligned}$$

We relegate the calculations to the “Appendix A.1”, but we conclude

44 $$\begin{aligned} \left\langle -dU(\sigma _c,\sigma _p),(\sigma _c-1, \sigma _p-1) \right\rangle \ge C_1\left\| \sigma _c \right\| _2^2 + C_2\left\| \sigma _p \right\| _2^2 - W(\sigma _c,\sigma _p) \end{aligned}$$

where *W* is uniformly bounded on $P_{2,\mu }^2$, and $C_1,C_2$ strictly positive. Recall that constraining the problem to $P_{2,\mu }$ is equivalent to $\left\| \sigma _c \right\| _1 = 1, \left\| \sigma _p \right\| = 1$. Hence Eq. (44) tends to infinity as $\left\| (\sigma _c,\sigma _p) \right\| _2$ tends to infinity. Therefore Eq. (43) is only negative on a bounded subset of $P^2_{2,\mu }$, showing existence of a solution to the variational inequality defined by the function Eq. (42) by Theorem 3.

To show strict pseudomonotonicity, we again apply Lemma 1. Assume that

45 $$\begin{aligned} \left\langle -dU((\sigma _c,\sigma _p)),(h_1, h_2) \right\rangle = 0 \end{aligned}$$

Re-arranging gives:

46 $$\begin{aligned} \frac{\varepsilon F_p N_c \left\langle \beta _p \sigma _c,h_2 \right\rangle }{(F_p + N_c\left\langle \beta _p \sigma _c,\sigma _p \right\rangle )^2} = c\left\langle \beta \sigma _p,h_2 \right\rangle N_p + \left\langle \frac{\sigma _c}{K_0 + K \phi },h_1 \right\rangle + \frac{F_p N_p \left\langle \beta _p \sigma _p,h_1 \right\rangle }{(F_p + N_c\left\langle \beta _p \sigma _c,\sigma _p \right\rangle )}\nonumber \\ \end{aligned}$$

Introducing $\langle x |$ as the functional defined by the inner product with *x*, we calculate:

47 $$\begin{aligned} H(x)= & {} (\nabla -dU)(x)\nonumber \\= & {} \begin{bmatrix} \frac{N_c}{K} - \frac{F_p N_c N_p \langle \beta _p \sigma _p | \langle \beta _p \sigma _p |}{(F_p + N_c \left\langle \sigma _c,\beta _p \sigma _p \right\rangle )^2} &{} \frac{F_p^2 N_p \langle \beta _p \sigma _p |}{(F_p + N_c \left\langle \beta _p\sigma _c,\sigma _p \right\rangle )^2} \\ \frac{\varepsilon N_cF_p^2 (N_c \langle \beta _p \sigma _p | \beta _p - \langle \beta _p \sigma _p | F_p)}{(F_p + N_c \left\langle \beta _p \sigma _c,\sigma _p \right\rangle )^3} &{} \frac{\varepsilon N_c^2 F_p^2 \langle \beta _p \sigma _c | \langle \beta _p \sigma _c |}{(F_p + N_c \left\langle \beta _p \sigma _c,\sigma _p \right\rangle )^3}+c N_p \langle \beta _p | \end{bmatrix} \end{aligned}$$

We need to show that $\left\langle H(x)h,h \right\rangle >0$. We immediately see that the negative contribution from the lower-left corner is cancelled by the upper-right corner. Inserting the relationship Eq. (46) in the term from the lower right right corner in $\left\langle H(x)h,h \right\rangle$ allows cancellation of the negative terms from the upper left corner in $\left\langle H(x)h,h \right\rangle$. This shows the desired result. $\square$

#### Remark 3

From the proof of existence in Proposition 3, we can extract that a negative density dependence described by a quadratic form is enough for existence of a Nash equilibrium in a population as long as all other terms have sub-quadratic growth.

As we are interested in the fixed-points of the population dynamics Eq. (27), we show that a fixed-point of the population dynamics exists and is unique.

#### Theorem 7

The population game Eq. (27) has a unique co-existence fixed point.

#### Proof

The stationary-point mapping of the behaviorally modified Rosenzweig–MacArthur system is clearly continuous as a function of $\sigma _c,\sigma _p$. Due to the metabolic terms and logistic terms the set of fixed-points of is uniformly bounded in $\overline{\sigma _c}, \overline{\sigma _p}$, and non-empty for sufficiently large *K*. By Proposition 3 the Nash equilibrium exists and is unique for every $N_c, N_p$. The operator $(-f_c, -f_p)$ can be shown to be pseudomonotone in an entirely analogous fashion as $(-dU_c, -dU_p)$, and we omit the calculations. Therefore, by Theorem 5 any coexistence equilibrium for the population game is unique and this will exist for *K* sufficiently large. Hence the equilibrium is unique as desired. $\square$

### Parameters

We parametrize the model according to Kleibers’ law (Yodzis and Innes 1992), hence that clearance rates, metabolic loss and the maximal consumption rate all scale with the mass to the power of 0.75. We decompose the depth-dependent predator clearance rate into a constant and a depth-dependent function *D*(*x*). Denoting the consumer mass by $m_c$ and the predator mass by $m_p$, the parameters of the model are given by:

48 $$\begin{aligned} F_p= & {} \alpha m_p^{0.75} \nonumber \\ \beta _l(x)= & {} b m_p^{0.75} D(x) \nonumber \\ \beta _c= & {} b m_c^{0.75} \nonumber \\ \mu _p= & {} \gamma m_p^{0.75} \end{aligned}$$

We model light decay *I*(*x*) throughout the water column as $I(x)=\exp (-kx)$, hence the depth-dependent carrying capacity as following the light-curve:

49 $$\begin{aligned} \phi (x) = \exp (-kx) \end{aligned}$$

And the depth-dependent predator clearance rate as being specialized in hunting near the top of the water-column:

50 $$\begin{aligned} D(x) = \exp (-k/m \cdot x^2) \end{aligned}$$

The scaling parameters for the model are taken from Andersen (2019, Table 2), except for the zooplankton mass which is from Kiørboe (2011).

**Table:** 

Name	Value	Meaning
$m_c$	0.01 g	Consumer mass
$m_p$	10 g	Predator mass
$\alpha$	1.25 $\hbox {g}^{1/4}$/month	Scaling of consumption rate
*b*	27.5 $\hbox {g}^{1/4}$ $m^3$/month	Scaling of clearance rate
$\gamma$	0.2	Ratio between max growth and respiration
$K_0$	$10^{-4}$ g $\hbox {m}^3\cdot$month	Minimal carrying capacity
$\beta _{p,0}$	$10^{-4}$ $\hbox {m}^3$/month	Minimal predator clearance rate
$\mu _p$	0.35 $\hbox {g}/(\hbox {m}^3 \cdot$ month)	Predator metabolic rate
$F_p$	7 $\hbox {g}/(\hbox {m}^3 \cdot$ month)	Predator maximum growth rate
$\varepsilon$	0.1	Trophic efficiency
*k*	0.05 $\hbox {m}^{-1}$	Light attenuation
$\kappa$	$\frac{1}{10}$ $\hbox {m}^{2}$	Decay of predation success

## Numerical approach and results

### Numerical implementation

In order to find Nash equilibria and fix-points of the behaviorally modified Rosenzweig–MacArthur system Sect. 4, we use the formulation of Eq. (10). We discretize space uniformly, using the trapezoidal rule to evaluate the integrals. By using the trapezoidal rule, we keep a banded sparsity pattern in the coupling of the locations. The equations Eq. (27) and the functions $-dU_c, -dU_p$ are formulated via. the symbolic language CasADi (Andersson et al. 2019), where we then solve the complementarity problem as a feasibility problem using IPOPT (Wächter and Biegler 2006) using the HSL subroutines for linear algebra (HSL 2007). We verified the numerical results by also solving the problem with a non-linear complementarity routine from the open-source package SICONOS (Acary et al. 2019).

The numerical approach for finding Nash equilibria and fixed points is extremely fast, and should scale to much larger problems. It allows for determination of fixed-points of the dynamics in less than 1 s with several hundred grid points. Simulating the population dynamics is, in contrast, a comparatively slow affair since we simulate the population dynamics using a forward Euler method.

### Population dynamics

With a numerical approach in place, we can perform numerical experiments to study the population dynamics and the impact of carrying capacity (*K*) and intraspecific predator competition (*c*) on the distributions and populations at equilibrium on the model in Sect. 4.

**Fig. 1:**
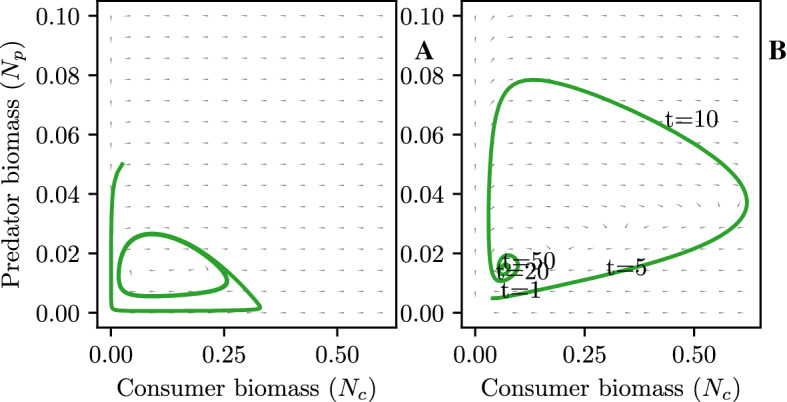
Phase portrait of the Rosenzweig–MacArthur system without optimal behavior $(\sigma _c = 1, \sigma _p = 1)$, (**A**) and with optimal behavior (**B**) at carrying capacity of $K=40$ and a competition of $c=0$. The green lines show a system trajectory

The direction of the flow with optimal behavior (Fig. 1B) is consistent with the usual Rosenzweig–MacArthur system (Fig. 1A). The phase portrait reveals that the system dynamics have been stabilized. Looking at the sample trajectory, the system has been been damped. The stable dynamics stand in contrast to the Rosenzweig–MacArthur model with constant behavior $(\sigma _p=\sigma _c=1)$ where the point of the Hopf bifurcation has been passed (Rosenzweig 1971), leading to limit cycles (Fig. 1).

**Fig. 2:**
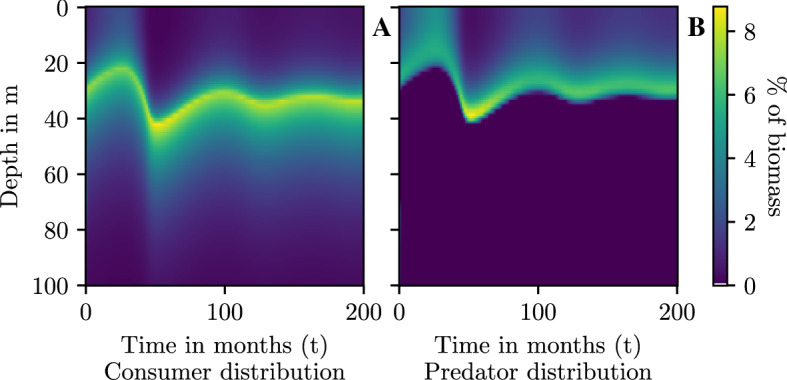
Transient strategies of consumers (**A**) and predators (**B**) at carrying capacity of $K=40$ and a competition of $c=0$ corresponding to the phase portrait Fig. 1

Both consumer and predator strategies change rapidly at the start of the time-interval, before stabilizing towards the equilibrium values (Fig. 2). It appears that the consumers are more present in the most productive area when the predator population is lower (Fig. 2A), which is not that surprising.

**Fig. 3:**
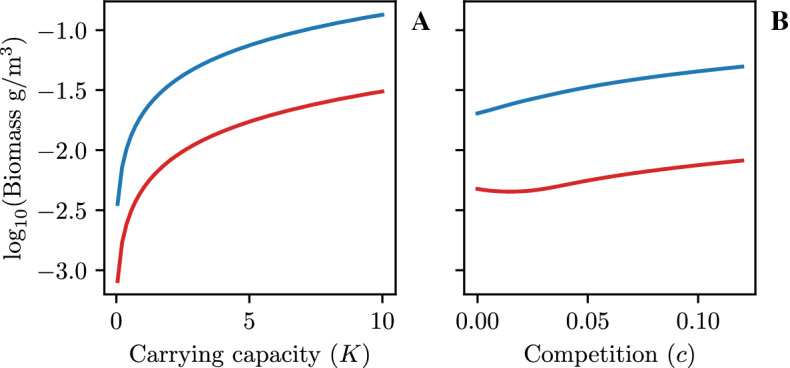
Panel (**A**) shows population levels of consumers (blue) and predators (red) at equilibrium with changing carrying capacity (*K*). Panel (**B**) again shows the population levels, but with varying intraspecific predator competition (*C*)

### Population at equilibrium

Figure 3 reveals how the population levels of consumers and predators change at equilibrium with varying carrying capacity (Fig. 3A) and intraspecific predator competition (Fig. 3B).

A higher carrying capacity causes higher populations of both consumers and predators populations at equilibrium (Fig. 3A). The increase in both populations is probably because the behavioral choice allows the consumers to avoid the risk of predation, while achieving the same fitness.

Varying the intraspecific predator competition causes an increase in the population of predators (Fig. 3B, red) until a point where the population stabilizes (Fig. 3($c\approx 1/3$)). The population of consumers continues to increase (Fig. 3B, blue) throughout.

### Spatial distributions

We start by investigating the spatial distribution of consumers and predators compared to their spatially varying fitness ($-dU_c,~-dU_p$).

**Fig. 4:**
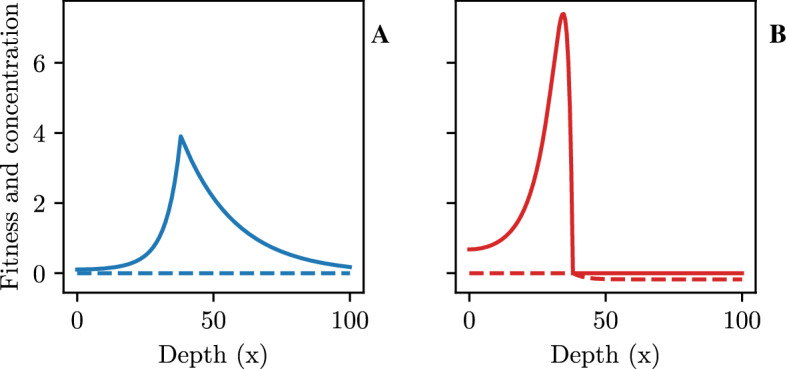
Spatial distribution (full lines) and fitness (dashed lines) of consumers (**A**) and predators (**B**) at the equilibrium with carrying capacity $K = 3$

Both consumers and predator distributions have a constant fitness of zero in the area with coexistence, where the fitness of the predators changes when their concentration is zero. In this we recognize the emergence of the ideal free distribution (Fig. 4B).

**Fig. 5:**
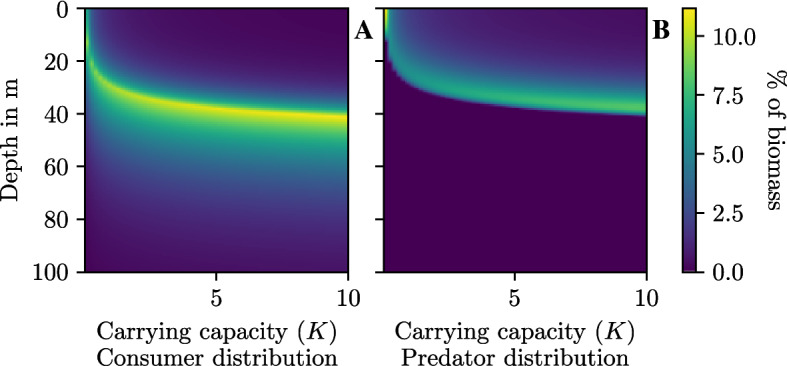
Spatial distribution of consumers $({\textbf {A}})$ and predators $({\textbf {B}})$ at the equilibrium with increasing carrying capacity (*K*)

At low carrying capacity consumers are relatively spread out in the most optimal part of the habitat (0$-$0.3), while predators are concentrated near the most optimal part (0). As the carrying capacity increases, the distribution of consumers becomes more concentrated, distributed around a peak of 0.4. The peak slowly moves downward with increasing carrying capacity. The consumers can be found throughout the habitat, even at the points of lowest productivity.

Predators go from being concentrated to very spread out, but surprisingly the peak of the predator distribution is just above the peak of the consumer distribution. There are no predators below the band of highly concentrated consumers. This is quite surprising since they have a non-zero encounter rate everywhere. The predator and consumer distributions follow each other as the carrying capacity increases, and appear to approach a stable asymptote (Fig. 5).

**Fig. 6:**
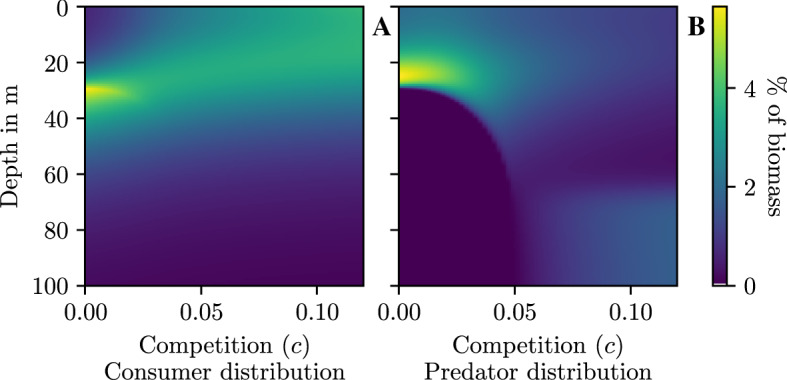
Distribution of consumers $({\textbf {A}})$ and predators $({\textbf {B}})$ at equilibrium under changing predator competition (*c*)

When there is no intraspecific predator competition consumers are highly concentrated at about 0.4, while the predator distributions spreads from 0.4 to 0. The distribution of predators spreads out as we increase competition, before concentrating in the safest zone (1) again (Fig. 6B). The foraging benefits from clustering on the consumers is outweighed by the risk of encountering other predators. The movement of predators is echoed by the consumers. The consumers spread out and gradually migrate to the most productive area (0) (Fig. 6A). The spreading out of the consumer population though the predator population is concentrated far away is caused by the intraspecific competition between consumers, akin to the ideal free distribution. It appears that both consumer and predator densities are converging to asymptotic densities (Fig. 6).

## Discussion and conclusion

We study population games through the introduction of mean-field games, which generalize the ideal free distribution (Fretwell 1969) to multi-species settings, albeit without the dynamical considerations of the multi-species ideal-free distribution (Cressman and Křivan 2010). We establish existence and uniqueness of Nash equilibria for a large class of population games using variational inequalities. In particular, we are able to handle a wide class of payoff functions with unique extrema and continuous strategy spaces. Having determined existence and uniqueness of Nash equilibrium for the instantaneous game, we showed the existence and uniqueness of fixed-points for suitably nice population games. This provides a simple criterion for population games, extending theorems based on specific models (Cressman and Křivan 2010; Sandholm 2010). As such, our work provides a multi-species generalization of the work on two-species ideal free distributions (Cressman and Křivan 2010; Cressman et al. 2004) and provides a generalization of the criteria for a unique equilibrium in a habitat selection game (Cressman and Křivan 2006, Appendix B).

We demonstrate the utility of our results by applying them to study a Rosenzweig–MacArthur system with fast optimal behavior. We establish existence and uniqueness of Nash equilibria, both for only consumers or predators and when both have optimal behavior. The method of proof is computational, and hence can almost certainly be extended to larger more complex ecosystems where the Nash equilibrium appears unique but has not been shown to be unique (Pinti et al. 2019). This shows that our general results open up the study of population games from a general mathematical viewpoint than has otherwise been the case (Cressman and Křivan 2010; Křivan 2013; Krivan and Cressman 2009; Broom and Rychtár 2013).

After showing existence and uniqueness, we analyzed the modified Rosenzweig–MacArthur game numerically by discretizing space. Adding optimal individual behavior appears to eliminate the paradox of enrichment (Rosenzweig 1971), which is a common consequence of optimal behavior in ecosystem models (Abrams 2010). We were unable to find a Lyapunov function to provide a theoretical justification (Krivan and Cressman 2009). In the sensitivity analysis we saw that the intraspecific predator competition did not noticeably affect the predator population levels, while elevating the consumer population levels, which was surprising (Abrams 2010). The increase in carrying capacity increased both predator and prey levels, as is usually the case in models with optimal behavior (Valdovinos et al. 2010). The numerical analysis also showed the emergence of an interesting pattern of consumer predator co-existence, with an ideal-free distribution emerging in the areas without any predators. In our numerical experiments we saw that changing the predator competition had a powerful indirect on both distribution and population of prey. The ecological interest of these results is supported by corresponding effects appearing when movement is not instantanenous and information is limited (Flaxman et al. 2011).

Our definition of an evolutionarily stable strategy (ESS) follows (Cressman et al. 2001), but generalized to function spaces. This definition allows for verification of whether a Nash equilibrium is an ESS, without taking population dynamics into account (Cressman and Křivan 2010). Though the definition does directly draw on population dynamics, whether a Nash equilibrium constitutes an ESS can be tested by studying the population dynamics (Grunert et al. 2021). This method of attack may reveal greater insights on the coupling of the population dynamics and the inner game, but is computationally heavy.

The key assumption in our modeling approach is the of instantaneous optimal behavior. Instantaneous optimal behavior in a transient population is reasnoable model if there is a decoupling between behavioral and population-dynamical time-scales. If this decoupling is not present, then the populations cannot be expected to follow the simple ideal free distribution at transient states (Abrams et al. 2007; Lou et al. 2014). The evolutionary stability of strategies leading to the simple ideal free distribution can break down, for instance when migrations driven by diffusion (Cantrell et al. 2010), or the resources and interactions are too irregular (Averill et al. 2012). As such, the model of instantaneous optimal behavior must be used with care, but is particularly suited for studying populations at steady-state (Cantrell et al. 2020, 2010, 2012a, b) or populations with separate behavioral and population-dynamical time-scales (Cressman and Křivan 2006; Křivan 2013).

Though the instantanenous ideal free distribution may serve to stabilize the dynamics, this is not always the case when the population dynamics and migration dynamics cannot be modeled on separate time-scales. When the simple ideal free distribution emerges through an explicit advection–diffusion model in a two-species setting, the simple ideal free distribution can serve to destabilize the population dynamics with a slightly sub-optimal strategy leading to stable population dynamical regime (Zelenchuk and Tsybulin 2021). Showing stability in systems with optimal behavior like the behaviorally modified Rosenzweig–MacArthur system is a hard analytical problem (Krivan and Cressman 2009). It seems a general approach could be drawing on the rapidly developing theory of dynamical variational inequalities (Adly 2018; Brogliato and Tanwani 2020; Tang et al. 2020) or studying dynamical systems associated to bi-level variational inequalities (Anh and Hai 2021). This could also provide a general theory of why optimal behavior generally enhances stability (Valdovinos et al. 2010). It appears that using these tools could be a promising future direction of research.

We have not touched on the topic of differential games, where the optimization is not instantaneous but takes e.g. the entire life-history into account. Variational inequalities can be applied to differential games (Pang and Stewart 2008), so this seems like a tantalizing next step. This could also provide a logical coupling with advection–diffusion dynamics to study e.g. habitats which are periodic in time (Cantrell et al. 2021).

By introducing mean-field games and studying them through variational inequalities, we show that it is possible to model the distribution of coexisting animal populations where all seek to optimize their foraging in models with strong time-scale separation or at the fixed-point. This enables accurate modeling of the spatial distribution of animals along with their populations, which moves us closer to the ultimate goal of being able to model the spatial distribution of animals exactly (Morris 2003).

## Data Availability

All data can be generated using the files 2d_sensitivity.py and phase_wrapper.py at https://github.com/jemff/MFG_Static.

## Calculations

### Existence

We complete the omitted calculations from the main text. Initially,

51 $$\begin{aligned} \left\langle -dU(\sigma _c,\sigma _p),(\sigma _c-1, \sigma _p-1) \right\rangle= & {} \left\langle \frac{N_c\sigma _c}{K_0 + K\phi } - 1,\sigma _c - 1 \right\rangle \nonumber \\&+ \left\langle \frac{F_p N_p\beta _p\sigma _p}{F_p + N_c\left\langle \beta _p\sigma _p,\sigma _c \right\rangle },\sigma _c-1 \right\rangle \nonumber \\&- \left\langle \frac{\varepsilon F_p^2N_c\beta _p\sigma _c}{(F_p + N_c\left\langle \beta _p\sigma _p,\sigma _c \right\rangle )^2},\sigma _p - 1 \right\rangle \nonumber \\&+ \left\langle N_p c \beta _p \sigma _p,\sigma _p -1 \right\rangle \end{aligned}$$

Using that $\left\langle 1,\sigma \right\rangle = 1$, we can write out Eq. (51) and gather the positive and negative terms

52 $$\begin{aligned}&\left\langle -dU(\sigma _c,\sigma _p),(\sigma _c-1, \sigma _p-1) \right\rangle \nonumber \\&\quad =\left\langle \frac{N_c\sigma _c}{K_0 + K\phi },\sigma _c \right\rangle + \left\langle N_p c \beta _p \sigma _p,\sigma _p \right\rangle \nonumber \\&\qquad + \left\langle \frac{F_p N_p \beta _p\sigma _p}{F_p + N_c\left\langle \beta _p\sigma _p,\sigma _c \right\rangle },\sigma _c \right\rangle - \left\langle \frac{\varepsilon F_p^2 N_c\beta _p\sigma _c}{(F_p + N_c\left\langle \beta _p\sigma _p,\sigma _c \right\rangle )^2},\sigma _p \right\rangle \nonumber \\&\qquad - \left\| N_p c\beta _p \sigma _p \right\| _1 - \left\| \frac{N_c \sigma _c}{K_0 + K\phi } \right\| _1 - \left\| \frac{N_p F_p\beta _p\sigma _p}{F_p + N_c \left\langle \beta _p\sigma _p,\sigma _c \right\rangle } \right\| _1 \nonumber \\&\qquad - \left\| \frac{\varepsilon F_p^2 N_c\beta _p\sigma _c}{(F_p + N_c\left\langle \beta _p\sigma _p,\sigma _c \right\rangle )^2} \right\| _1 \end{aligned}$$

To handle Eq. (52), we consider the individual terms. We start by considering the terms:

53 $$\begin{aligned}&\left\langle \frac{F_p N_p\beta _p\sigma _p}{F_p + N_c\left\langle \beta _p\sigma _p,\sigma _c \right\rangle },\sigma _c \right\rangle , \nonumber \\&\left\langle \frac{\varepsilon F_p^2 N_c \beta _p\sigma _c}{(F_p + N_c\left\langle \beta _p\sigma _p,\sigma _c \right\rangle )^2},\sigma _p \right\rangle \end{aligned}$$

We see both terms in Eq. (53) are uniformly bounded in $(\sigma _c,\sigma _p)$ over $P_{2,\mu }^2$, hence so is their difference $W_0(\sigma _c,\sigma _p)$. Defining $C_1 = \frac{1}{K_0 + K {{\,\mathrm{ess}\,}}\sup \phi }$ and $C_2 = c {{\,\mathrm{ess}\,}}\inf \beta _p$ we can rewrite Eq. (52) as:

54 $$\begin{aligned} \begin{aligned} \left\langle -dU(\sigma _c,\sigma _p),(\sigma _c-1, \sigma _p-1) \right\rangle \ge C_1 \left\| \sigma _c \right\| ^2_2 + C_2 \left\| \sigma _p \right\| _2^2 \\ - \left\| c\beta _p \sigma _p \right\| _1 - \left\| \frac{\sigma _c}{K_0 + K\phi } \right\| _1 - \left\| \frac{F_pN_p\beta _p\sigma _p}{F_p + N_c\left\langle \beta _p\sigma _p,\sigma _c \right\rangle } \right\| _1 \\ - \left\| \frac{F_p^2 N_c\beta _p\sigma _c}{(F_p + \left\langle \beta _p\sigma _p,\sigma _c \right\rangle )^2} \right\| _1 + W_0(\sigma _c,\sigma _p) \end{aligned}\nonumber \\ \end{aligned}$$

Since $\left\| \sigma _i \right\| _1=1, i \in \{c,p\}$, all terms involving $\left\| \cdot \right\| _1$ in Eq. (52) are uniformly bounded, and can be gathered with $W_0$ in a single uniformly bounded function *W*. Hence we end with:

55 $$\begin{aligned} \left\langle -dU(\sigma _c,\sigma _p),(\sigma _c-1, \sigma _p-1) \right\rangle \ge C_1 \left\| \sigma _c \right\| _2^2 + C_2 \left\| \sigma _p \right\| _2^2 - W(\sigma _c,\sigma _p) \end{aligned}$$

## Declarations

### Code availability

All code for reproducing the results of this project is available on github https://github.com/jemff/MFG_Static.
